# Investigating the Effect of Biomaterials Such as Poly-(l-Lactic Acid) Particles on Collagen Synthesis In Vitro: Method Is Matter

**DOI:** 10.3390/jfb11030051

**Published:** 2020-07-24

**Authors:** Subarna Ray, Hang T. Ta

**Affiliations:** 1 Australian Institute for Bioengineering and Nanotechnology, University of Queensland, Brisbane, QLD 4072, Australia; subarna.ray@uq.net.au; 2 Queensland Micro- and Nanotechnology Centre, Griffith University, Brisbane, QLD 4111, Australia; 3 School of Environment and Science, Griffith University, Brisbane, QLD 4111, Australia

**Keywords:** collagen, biomaterials, poly-(l-lactic acid), fibroblast, macrophage

## Abstract

Poly-l-lactic acid (PLLA), a synthetic, biocompatible, biodegradable polymer, has been safely used in several clinical applications in recent decades. Typically, Sculptra^TM^, the commercially injectable PLLA in the form of microparticles, has been used as facial volumizer in the treatment of lipoatrophy in HIV patients. It also has various applications in tissue engineering by improving cell proliferation and adhesion. Sculptra™ can be categorised as a stimulatory filler as it stimulates the synthesis and deposition of fibrous tissue and collagen. Collagen is one of the most significant components of the extracellular matrix and beneficial for the normal physiology. It is also the structural component of a human body. In most of the studies, the effect of Sculptra on collagen synthesis was investigated in vivo and the majority of the data were from clinical and histological reports. There is only one study reporting this effect in vitro using fibroblasts. Here, we investigated whether PLLA in the form of nanoparticles can provide the same effect on collagen synthesis in fibroblasts as Sculptra. We surprisingly found that there was no stimulation of collagen in fibroblasts alone, whereas the co-cultures of fibroblast and macrophage had shown collagen stimulation by PLLA nanoparticles. It is also confirmed that collagen synthesis was caused by fibroblasts but not macrophages. Although further study needs to be conducted to evaluate its mechanism, our findings showed that choosing an appropriate method is essential for investigating the effect of PLLA or other biomaterials on collagen synthesis by fibroblasts in vitro.

## 1. Introduction

Sculptra (poly-(l-lactic acid) (PLLA) is a biocompatible and biodegradable polymer and has wide applications in wound healing, implants, dermatological treatment and medical devices. PLLA is preferred as fracture fixatures as it provides strength for bone healing [1]. VICRYL™ (PLA/PGA copolymer) is the commercially available material used in clinical applications. For the treatment of facial lipoatrophy, Sculptra™ (PLLA microparticles) is used as a facial volumizer and is approved by the FDA [2]. It increases the collagen by stimulating the fibroblasts via a foreign body reaction and thickens the dermis by acting as a dermal matrix [3]. Therefore, a wide application of PLLA has been explored and sets as a potential material in various domains of applications in the future. Sculptra can be categorized as a stimulatory filler and it is different from traditional fillers as it does not take up space but causes the gradual deposition of fibrous tissue or collagen for volume restoration [4]. Collagen buildup occurs over a series of injections and at least three sessions are necessary for the optimal results. PLLA gradually reabsorbs once the results occur. This approach can be used for a long-term result and over a large surface area. It is used in common areas, such as cheeks, temples, inframalar region, chin, prejowl sulcus, marionette lines, infraorbital regions and lateral brow. According to a study by the Sculptra cosmetic trial group, continued progression of significant improvements was seen in the nasolabial fold out to 13 months [5].

Sculptra has been used in major clinical studies. An example is a study that was conducted in HIV-related facial lipoatrophy patients over a period of 2 years. The patients were injected every 2 weeks completing four to five injections. A significant three-fold increase in the skin thickness was observed, which sustained over 2 years [6]. The mechanism of action is thought to be a stimulation of a foreign-body reaction characterized by increased macrophages, mast cells, and lymphocytes. This influx leads to slow degradation of the product-increased fibroblastic activity and gradual neocollagenesis [7]. Werschler et al. identified increased levels of type I collagen in histologic studies of treated skin [8]. New collagen appears to form by 1 month and continues to increase for 9 months to a year. The PLLA particles show signs of breaking down around 6 months and are gone by 9 months. There are a number of clinical and histological reposts showing the effect of Sculptra in vivo [9,10,11]. There is only one study by Kim et al. in 2019 reporting the effect of Sculptra on collagen synthesis by fibroblasts (single culture) in vitro [12].

The general aim of this study is to investigate whether PLLA materials in nanoscale are able to stimulate collagen synthesis. PLLA nanoparticles were synthesized and their effect on collagen synthesis was studied in vitro using a single cell culture of fibroblasts and a co-culture of fibroblasts and macrophages. Surprisingly we found that a single culture of fibroblasts could not enhance collagen synthesis, whereas a co-culture of fibroblasts and macrophages could.

## 2. Materials and Methods

### 2.1. Cell Culture

HFF1 (ATCC^®^SCRC-1041™) fibroblast cells were cultured in T75 flasks using ATCC-formulated Dulbecco’s Modified Eagle’s Medium (DMEM) supplemented with 15% fetal bovine serum and 1% penicillin streptomycin antibiotic. Macrophages J774A.1 (ATCC^®^ TIB-67™) were cultured in a Petri dish using ATCC-formulated RPMI medium (Roswell Park Memorial Institute medium) as the base medium supplemented with 10% fetal bovine serum and 1% penicillin streptomycin antibiotic. The flasks were incubated under 37 °C and 5% CO_2_ condition.

### 2.2. Nanoparticle Preparation and Characterization

PLLA nanoparticles were prepared using the solvent evaporation method in an oil-in-water emulsion. PLLA polymer (0.08% *w*/*w*) was dissolved in dichloromethane (DCM) as the organic solvent. One percent PVA, poly (vinyl alcohol), was used as the stabilizer for PLLA nanoparticles. The polymer dissolved in DCM was injected into the stabilizer solution by Adelab New Era NE-8000 high pressure syringe pump^®^ at an injection rate of 150 µL/min under a magnetic rotator at 600 rpm. The mixture was homogenized using Ultra-TURRAX^®^ T25 basic IKA^®^ WERKE homogenizer at a speed of 6500 rpm during the injection of the polymer and an additional 5 min. The suspension was then probe sonicated using ultrasonic processor (SONIC Vibra cells^®^) at an amplitude of 60 (2 watts) for 10 min on ice. The DCM in the suspension was evaporated by heating at 50 °C for 1 h. The nanoparticles were washed by centrifugation at 10,000× *g* for 30 min twice and resuspended in MilliQ water. To estimate the concentration of the nanoparticle stock, a sample of 50 µL was freeze-dried overnight and weighed and the concentration was calculated. The nanoparticle size was determined and characterized by using Zetasizer 7.12.

### 2.3. Cell Viability Assay

Macrophages (J774A.1) and Fibroblasts (HFF1) were plated on separate 96-well plates at 10,000 cells/well. After 24 h of incubation at 37 °C in 5% CO_2_ concentration, the wells were treated with the following PLLA nanoparticle concentrations: 0, 10, 30, 100, 300, 1000 µg/mL and the cells were incubated for another 24 h at 37 °C in 5% CO_2_ concentration. Cell viability was measured by Presto Blue™ cell viability reagent (Invitrogen) according to manufacturer protocol. Briefly, cells were washed with 1x PBS (pH 7.4) and then 100 µL of 10% Presto Blue solution was added into each well. An additional well was filled with 10% Presto Blue solution, which acts as the background control. The 96-well plates were then incubated at 37 °C in 5% CO_2_ concentration for 30 min and the fluorescence readings were recorded at an excitation wavelength of 535 nm and an emission wavelength of 615 nm. The readings were subtracted from the background control and the graphs were plotted using GraphPad Prism 8. Each sample concentration was done in triplicates.

### 2.4. PLLA Treatment on Fibroblast and Macrophage Single Cultures

Fibroblasts (HFF1 (ATCC^®^SCRC-1041™)) were seeded at a concentration of 150,000 cells per well in a 6-well plate. After 24 h, fibroblasts were treated with different PLLA nanoparticle concentrations (30, 100 and 300 µg/mL), 1 mg/mL Sculptra™ for 24 h at 37 °C and 5% CO_2_ concentration. A total of 100 ng/mL of TGFβ was used as a positive control for collagen stimulation.

Similarly, macrophages J774A.1 (ATCC^®^ TIB-67^™^) were seeded at a concentration of 250,000 cells per well in a 6-well plate. After 24 h, the cells were treated with 100 µg/mL of PLLA nanoparticles to investigate any significant collagen stimulation by macrophages. This concentration was chosen because significant collagen stimulation was observed at this particular concentration in fibroblast/macrophage co-culture.

### 2.5. PLLA Treatment on Fibroblast/Macrophage Co-Culture

Co-cultures of fibroblasts and macrophages were prepared in a 6-well plate by seeding fibroblasts followed by macrophages. Fibroblasts (HFF1) were first coated in a 6-well plate at a cell density of 150,000 cells per well and incubated at 37 °C with 5% CO_2_ concentration for 24 h. After 24 h of incubation, macrophages J774A.1 were then seeded into the appropriate wells at a cell density of 250,000 cells per well to form co-cultures in DMEM growth medium, supplemented with 15% fetal bovine serum and 1% penicillin streptomycin antibiotic. After 24 h of incubation, the co-cultures were treated with different concentration of PLLA nanoparticles (30, 100 and 300 µg/mL) and Sculptra™ (Galderma Australia Pty Ltd., Sydney, Australia) at 1 mg/mL for 24 h.

### 2.6. Collagen Assay

The cells were collected in 1x PBS (pH 7.4) and centrifuged at 1000× *g* for 15 min and the cell pellet was suspended in cold 0.5 M acetic acid. The cell solution was homogenized on ice using a pre-chilled Dounce homogenizer. Following overnight incubation, the acidic solution was centrifuged at 10,000× *g* for 15 min at 4 °C. The supernatant was collected and neutralized using 0.5 M NaOH. Collagen concentration was measured using the Soluble Collagen Assay Kit^®^ (ab241015, Abcam, Cambridge, UK) according to manufacturer’s instruction. The fluorescence was measured at an emission wavelength of 468 nm and an excitation wavelength of 376 nm using a Kaleido™ microplate reader. The graphs were plotted using GraphPad Prism 8.

### 2.7. Statistics Analysis

The data was expressed as a mean (+/−) standard deviation for *n* = 3–4. Student’s t test was performed using GraphPad Prism 8. *p* < 0.05 was considered significant.

## 3. Results

### 3.1. PLLA Nanoparticle Characterization

**Figure 1:**
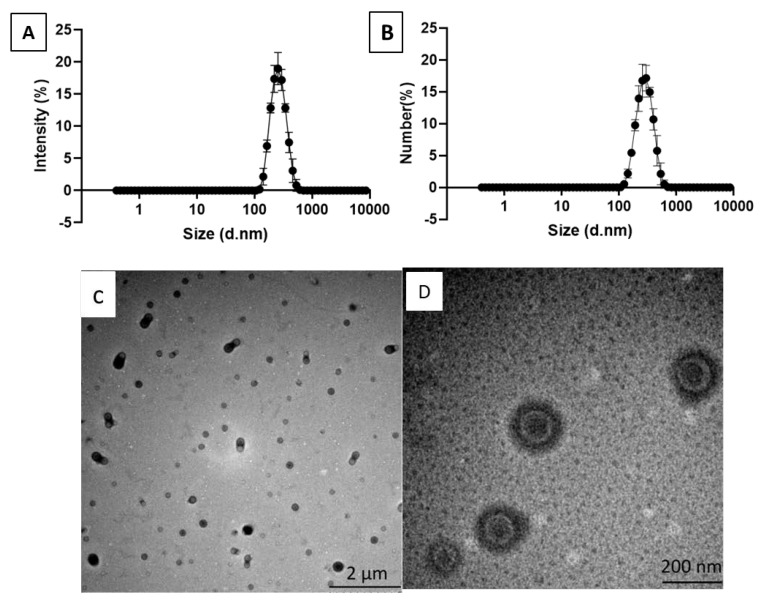
Characterization of the synthesized poly-l-lactic acid (PLLA) nanoparticles. (**A**) An intensity graph of PLLA nanoparticles; (**B**) a number graph of PLLA nanoparticles; (**C**) A TEM image of PLLA nanoparticles at low magnification (scale bar: 2 µm); (**D**) a TEM image of PLLA nanoparticles at high magnification (scale bar: 200 nm).

PLLA nanoparticles were prepared by the emulsion and solvent evaporation method and the size distribution of the nanoparticles was measured by dynamic light scattering (DLS). The nanoparticles had an average diameter (Z-average) of 248 ± 3 nm, number mean (Nm) of 224 ± 7 and a polydispersity index (PDI) of 0.075 ± 0.021 (Figure 1A,B). The zeta potential of the PLLA nanoparticles was 0.273 ± 0.201 mV. Figure 1C,D shows the TEM images of PLLA nanoparticles.

### 3.2. Viability of Macrophages and Fibroblasts Treated with PLLA Nanoparticles

**Figure 2:**
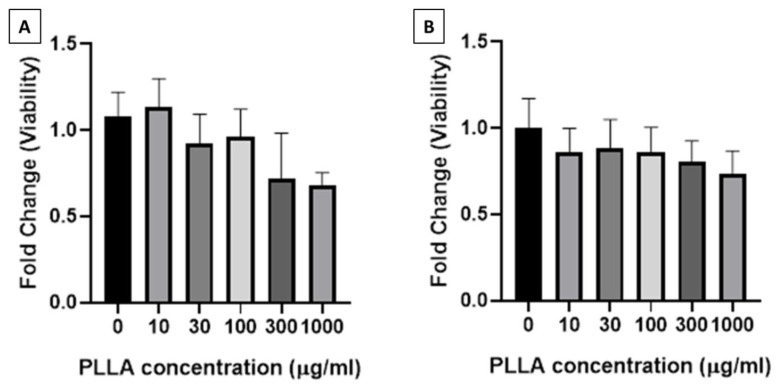
Viability of (**A**) macrophages (J774A.1) and (**B**) fibroblasts (HFF1) treated with PLLA nanoparticles at 0–1000 µg/mL for 24 h.

The viability of macrophages (J774A.1) and fibroblasts (HFF1) treated with PLLA nanoparticles decreased with the increase in the concentration of PLLA nanoparticles (Figure 2. Macrophage viability remained above 90% until 100 µg/mL of nanoparticles was used and reduced to 75% when treated with 300 µg/mL and 1000 µg/mL of nanoparticles. Similarly, fibroblast viability decreased minimally in a stepwise manner until the concentration of 1000 µg/mL was used. Therefore, PLLA nanoparticles at a concentration of up to 300 µg/mL were used in collagen assay, as this concentration is viable for both the cell lines.

### 3.3. Collagen Synthesis Stimulated by PLLA Nanoparticles

**Figure 3:**
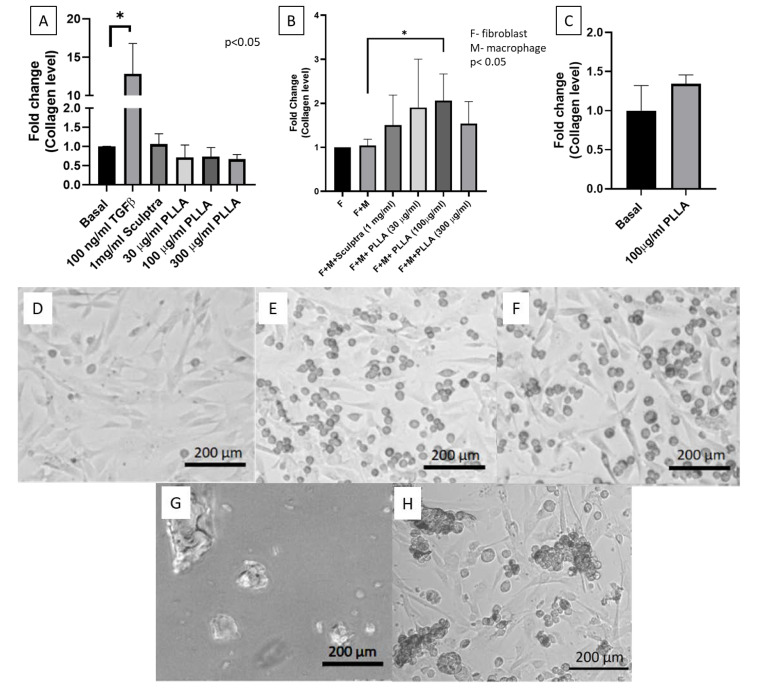
Collagen stimulation graphs and optical images of different cell types treated with PLLA nanoparticles. (**A**) A collagen stimulation graph of human fibroblasts (HFF1) treated with PLLA nanoparticle concentration (30, 100 and 300 µg/mL), Sculptra™ (1 mg/mL) and 100 ng/mL of TGFβ as a positive control for 24 h; (**B**) a collagen stimulation graph of fibroblast macrophage coculture when treated with PLLA nanoparticles with a concentration of 30, 100 and 300 µg/mL and Sculptra™ (1 mg/mL) for 24 h; (**C**) a collagen stimulation graph of macrophages (J774A.1) treated with PLLA nanoparticle concentration (100 µg/mL); (**D**) a fibroblast without PLLA nanoparticles, which acts as the baseline; (**E**) macrophage-fibroblast co-culture without any treatment (control); (**F**) macrophage-fibroblast co-culture treated with PLLA nanoparticles (30 µg/mL) for 24 h; (**G**) optical images of Sculptra™ (scale bar 200 µm); (**H**) macrophage morphology and the accumulation of macrophages around Sculptra™ in co-cultures treated with Sculptra™ for 24 h. * *p* < 0.05.

Human fibroblasts (HFF1) were treated with PLLA nanoparticles, TGFβ as a positive control, and Sculptra™ (commercially available PLLA microparticles) for 24 h. Cells treated with the positive control (100 ng/mL of TGFβ) showed more than 10-fold stimulation for collagen synthesis after 24 h of incubation. The cells treated with 1 mg/mL Sculptra™ did not show any stimulation for collagen synthesis. PLLA nanoparticles at all the concentrations tested (30, 100 and 300 µg/mL) did not show any significant stimulation for collagen synthesis (Figure 3A).

Since PLLA nanoparticles could not stimulate collagen synthesis in fibroblast single cultures, we attempted to test stimulation of collagen synthesis in fibroblast/macrophage co-cultures at the same PLLA nanoparticle concentrations (30, 100 and 300 µg/mL) and 1 mg/mL Sculptra™. A total of 1 mg/mL Sculptra was chosen as it was shown to stimulate collagen production by Kim et al. [12]. The collagen levels were measured after 24 h of particle treatment. Figure 3B shows that significant stimulation of collagen synthesis could be seen when the co-culture was treated with 100 µg/mL of PLLA nanoparticles and this particular concentration was viable for both types of cell lines (fibroblasts HFF1 and macrophages J774A.1). A higher concentration (300 µg/mL), however, did not significantly stimulate collagen synthesis, probably because of the lower cell viability at this concentration. Sculptra at 1 mg/mL also could not enhance collagen synthesis. This is probably due to the large size of Sculptra. Figure 3D–F shows the morphology of fibroblast alone, the co-culture of fibroblast and macrophage, and the co-culture treated with PLLA nanoparticles, respectively. While PLLA nanoparticles could not be seen in Figure 3F due to their small size, Sculptra particles were visible in Figure 3G,H. Figure 3G shows Sculptra microparticles with various shapes and sizes (ranging from 10–200 µm). The cell morphology and orientation when the co-culture of cells was treated with 1 mg/mL Sculptra™ can be seen in Figure 3H. Macrophages were found accumulating and clustering around the Sculptra™ microparticles. In contrast, PLLA nanoparticles were most likely taken by macrophages, which is evidenced by the presence of foam cells (Figure 3F). The uptake of PLLA nanoparticles by macrophages would be responsible for the stimulation of collagen synthesis in fibroblasts. Macrophages play a key role in stimulating the secretion of various chemokines and cytokines. TGFβ, a cytokine stimulating the collagen synthesis in fibroblasts, is secreted by macrophages by the action of a foreign body reaction.

To investigate the role of macrophages in the co-culture system and to confirm that macrophages did not produce collagen, leading to the high level of collagen detected in the co-culture system, we treated macrophages with 100 µg/mL of PLLA nanoparticles. Figure 3C shows that 100 µg/mL of PLLA nanoparticles did not significantly stimulate collagen synthesis as compared to the control (basal). Therefore, it was confirmed that collagen synthesis did not occur in macrophages when treated with 100 µg/mL of PLLA nanoparticles.

## 4. Discussion

Sculptra was found superior to conventional classic fillers in terms of stability and effect persistence. Type I collagen is significantly increased up to 6 months after injection of Sculptra under human skin [13]. Its clinically volume-augmenting effect could be seen even within a month. Collagen stimulation following injection with Sculptra has been explored in both animal models and human studies [9]. It was assumed that a weak subclinical inflammatory reaction occurred at the site of injection, leading to the infiltration of neutrophils, macrophages and then foreign body giant cells and elongated fibroblasts, followed by fibroplasia and resultant collagen type I deposition in the extracellular matrix [10]. Although a number of clinical and histological data were reported, the molecular biologic effects of PLLA have rarely been investigated. To our knowledge, the study from Kim et al. [12] is the only one that tried to explore the effect of Sculptra on collagen synthesis and related signaling pathways in cultured dermal fibroblast. However, collagen synthesis is a complex in vivo event. The simple single fibroblast culture in Kim et al. study is limited in providing insights into the collagen synthesis effects of Sculptra or PLLA microparticles.

In our study, we aimed to investigate whether PLLA particles in the nanoscale (instead of microscale) are able to induce the same collagen synthesis effect as Sculptra. We first followed Kim et al.’s approach where we used single fibroblast culture for the study. However, due to the complex nature of collagen synthesis in vivo and the involvement of inflammation reaction, we proposed to try co-culture of fibroblasts and macrophages. We found that human fibroblasts (HFF1) when cultured in vitro with 1 mg/mL Sculptra™ and PLLA nanoparticles (concentrations: 30, 100 and 300 µg/mL) did not show a stimulation in the collagen synthesis within 24 h of treatment. These results were consistent with Kim et al., where a stimulation in collagen synthesis was not seen when dermal fibroblasts were cultured in vitro with 0.1% Sculptra for 24 h [12]. However, when co-culture setting was employed, it was possible to see the effect on collagen stimulation within 24 h of treatment with PLLA nanoparticles. We hypothesized that the PLLA action on collagen synthesis follows a foreign body reaction and increases the collagen levels in fibroblast/macrophage co-culture.

A foreign body reaction in a fibroblast-macrophage co-culture can be an excellent model to study various cell to cell interactions. In our study, we are interested whether the phagocytosis of PLLA nanoparticles by macrophages are capable of stimulating collagen production in fibroblasts. Therefore, co-cultures of fibroblast and macrophages were used for this study. In the previous study, it was found that apoptotic bodies when ingested by macrophages caused a significant stimulation of collagen production in primary fibroblast in a co-culture [14]. Stimulation in collagen production occurs via secretion of various soluble factors produced by macrophages in response of any foreign body ingestion. This theory was proven by an experiment of co-cultures in contact as well as separated by trans-well membrane. A potent cytokine, TGFβ, secreted by macrophages is one of the factors responsible for collagen stimulation [15]. In our study, PLLA nanoparticles can be considered as a foreign body of a smaller size range and can therefore be up taken easily by macrophages, thus showing the effect on collagen synthesis quickly within 24 h. Sculptra, however, due to its large size, could not be taken by macrophages; thus, it was unable to provide a collagen stimulation effect in 24 h.

In our study, the higher concentration of PLLA nanoparticles (i.e., 300 µg/mL) reduced macrophage viability to 75% and failed to show any significant stimulation in collagen production in fibroblasts. The lower concentration of PLLA nanoparticles, on the other hand, failed to stimulate a sufficient level of TGFβ in macrophages for the stimulation of collagen production in fibroblasts. Polymeric nanoparticle cytotoxicity and viability have been tested on macrophages, and one such study shows that a concentration of 0.1 mg/mL poly(lactic-co-glycolic acid) (PLGA) nanoparticles has shown a higher concentration of cytokine production, whereas a lower concentration of PLGA nanoparticles had no secretion of cytokines [16].

## 5. Conclusions and Perspectives

Collagen is an important component of the extracellular matrix (ECM) along with other components of the ECM. Sculptra (PLLA microparticles) is a biodegradable and biocompatible polymer material used for facial volume enhancement by increasing collagen content over time and is demonstrated in vivo. This study aims to investigate the effect of PLLA nanoscale-particles on collagen synthesis. However, the collagen synthesis induced by PLLA is triggered by a range of foreign material responses and mild inflammation reactions. In this research, PLLA nanoparticles were synthesized and their effect on collagen synthesis was studied. PLLA is used in the form of nanoparticles, which are prepared by the solvent evaporation method. The concentration of PLLA used for the treatment in fibroblast and macrophage was determined according to the viability assays. PLLA fails to stimulate collagen production in fibroblast alone but this aim can be achieved by using macrophage-fibroblast co-culture at a concentration of around 100 µg/mL tested in vitro. The co-culture model is feasible to achieve this goal since macrophages are an integral component of an inflammatory reaction and the mechanism of action follows the foreign body reaction. Our study shows that fibroblast-macrophage co-culture can be an excellent model to study various cell to cell or biomaterial to cell interactions. It also proves that choosing an appropriate method is essential for investigating the effect of PLLA or other biomaterials on collagen synthesis by fibroblasts in vitro.
